# *In situ* accurate control of 2D-3D transition parameters for growth of low-density InAs/GaAs self-assembled quantum dots

**DOI:** 10.1186/1556-276X-8-86

**Published:** 2013-02-18

**Authors:** Mi-Feng Li, Ying Yu, Ji-Fang He, Li-Juan Wang, Yan Zhu, Xiang-jun Shang, Hai-Qiao Ni, Zhi-Chuan Niu

**Affiliations:** 1 State Key Laboratory for Superlattices and Microstructures, Institute of Semiconductors, Chinese Academy of Sciences, P. O. Box 912, Beijing 100083, China

**Keywords:** InAs quantum dots, Sacrificed InAs layer, Molecular beam epitaxy, Reflection high-energy electron diffraction, Micro-photoluminescence, Low density, 78.67.Hc, 78.55.Cr, 78.55.-m

## Abstract

A method to improve the growth repeatability of low-density InAs/GaAs self-assembled quantum dots by molecular beam epitaxy is reported. A sacrificed InAs layer was deposited firstly to determine *in situ* the accurate parameters of two- to three-dimensional transitions by observation of reflection high-energy electron diffraction patterns, and then the InAs layer annealed immediately before the growth of the low-density InAs quantum dots (QDs). It is confirmed by micro-photoluminescence that control repeatability of low-density QD growth is improved averagely to about 80% which is much higher than that of the QD samples without using a sacrificed InAs layer.

## Background

Single self-assembled semiconductor quantum dots (QDs) are of increasing interest due to their applications in low-threshold lasers [1], single-photon and entangled photon sources [2,3], quantum computing, and quantum information processing [4,5]. Several techniques have been developed to obtain low-density QD structures, such as the Stranski-Krastanov self-assembled growth of QDs on a substrate patterned with mesa/holes [6,7], stopping of the rotation of the substrate to obtain a gradient density of InAs QDs [8,9], and a modified droplet epitaxy method to lower the QDs' density [10]; especially one of the most effective method is to stop the InAs deposition at the onset of a two-dimensional to three-dimensional (2D-3D) growth transition [11] by controlling the parameters of 2D-3D growth transition such as temperature, growth rate, deposition amount of indium, and interruption time. However, the narrow range of deposition in the 2D-3D growth transition determines that allowed deviations of controllable parameters are quite limited for repeatable growth of low-density QDs.

In this paper, to increase the repeatability and to obtain good single-photon characteristics, we investigated a growth technique to obtain *in situ* the critical deposition in 2D-3D growth transition and slightly change the critical conditions to achieve InAs QDs with good single-photon characteristics. The success ratio is improved averagely to about 80% which is much higher than that of the traditional QD samples (less than 47%).

## Methods

**Figure 1:**
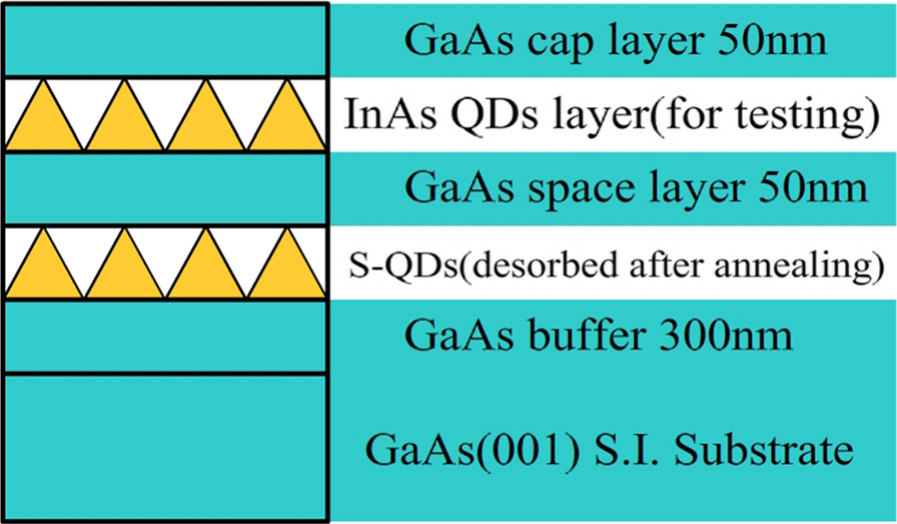
Schematic illustration of different deposition amounts of InAs on GaAs.

All the samples were grown using a Veeco Mod GIN II solid source MBE system (Veeco Instruments, Inc., Plainview, NY, USA). The sample structure is shown in Figure 1. A quarter of a 2-in. semi-insulating (100) GaAs wafer was kept under an As flux of 6 × 10^−6^ Torr beam equivalent pressure. A 300-nm undoped GaAs buffer layer was grown at a substrate temperature *T*_s_ of 580°C. A sacrificial InAs quantum dot (SQD) layer was deposited to confirm the critical condition of the 2D-3D growth transition *in situ* and then annealed immediately at 580°C, 590°C, and 610°C, respectively, for InAs desorption. After growing a 50-nm GaAs barrier layer to separate from the SQD layer, the growth temperature was lowered down to 520°C for the growth of InAs QDs, with a growth rate of 0.005 monolayer (ML)/s. A 50-nm GaAs capping layer and another similar QD layer were grown for the AFM test. All samples are displayed in Table 1. The critical coverage (*θ*_c_) was taken at the steep rise of the reflex intensity when the streaky pattern of the 2D wetting layer turned into the Bragg spots of the 3D QDs detected by reflection high-energy electron diffraction (RHEED) [12]. Fourier photoluminescence (PL) was excited by a 632.8-nm He-Ne laser at 80 K and detected by a liquid nitrogen-cooled CCD detector. Micro-PL used the confocal microscopy technique with a 2-μm-diameter laser spot. Transmission electron microscopy (TEM) was used to study the SQD and QD layers using a Tecnai F20 field emission gun transmission electron microscope (FEI Co., Hillsboro, OR, USA).

**Table 1:** Growth parameters of sample 1 to sample 9

**Samples**	**Growth temperature of SQD/QD (°C)**	**Growth rate (ML/s)**	**Deposition *θ***_**c**_ **+ Δ (ML)**	**Interruption time (s)**	**Annealing temperature (°C)**
1	520/525	0.005	*θ*_c_ + 0.15	10	610
2	520/525	0.005	*θ*_c_ + 0.075	10	610
3	520/525	0.005	*θ*_c_ + 0.025	10	610
4	520/525	0.005	*θ*_c_ + 0	10	610
5	520/525	0.005	*θ*_c_ − 0.05	10	610
6	520/525	0.005	*θ*_c_ − 0.075	10	610
7	520/525	0.005	*θ*_c_ + 0	10	580
8	520/525	0.005	*θ*_c_ + 0	10	590
9	-/525	0.005	*θ*_c_	10	-

There is no SQD and annealing step for sample 9.

## Results and discussion

**Figure 2:**
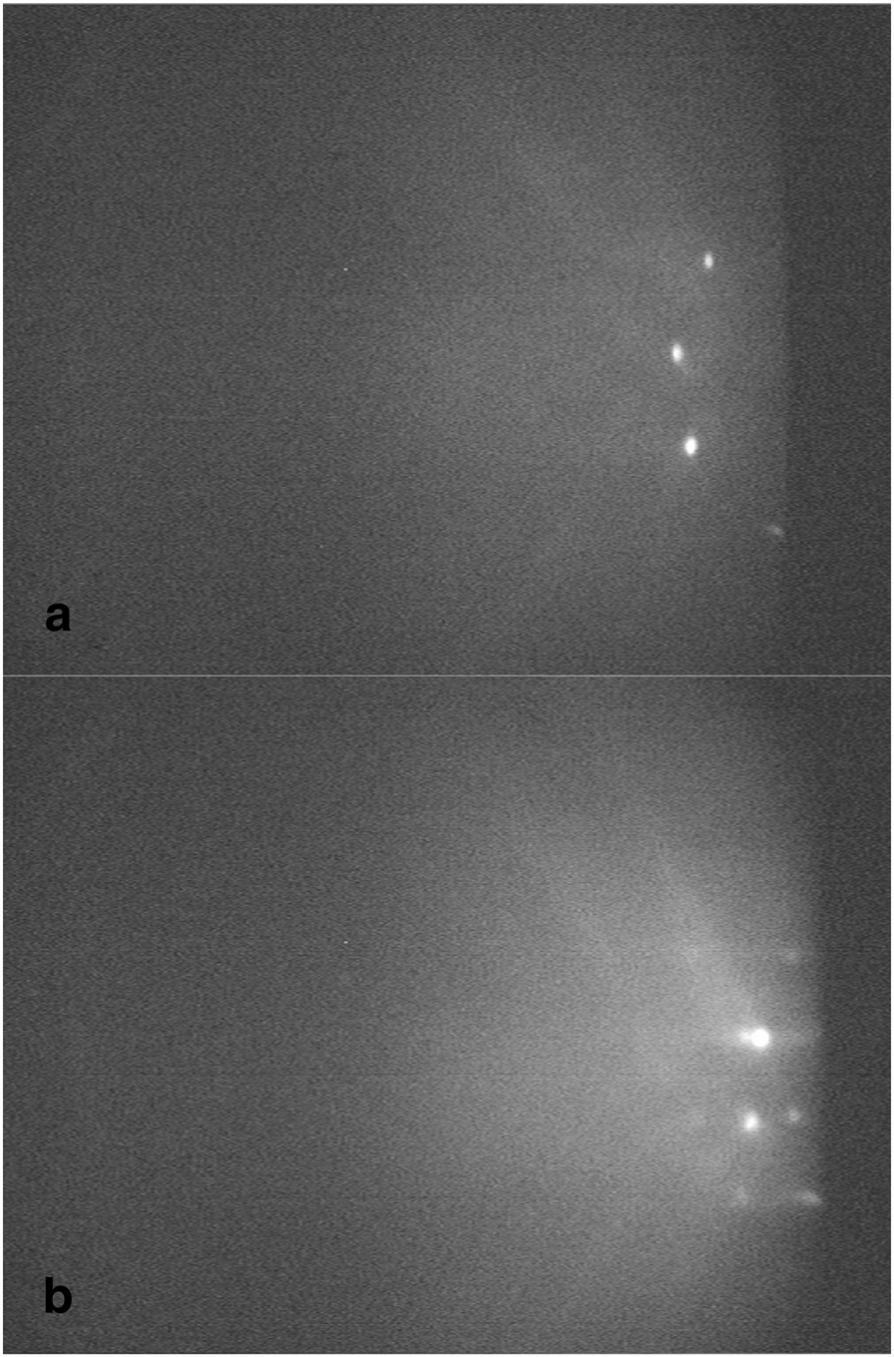
**RHEED patterns of InAs deposition.** (**a**) After deposition of InAs and before 3D growth and (**b**) when 2D-3D growth transition appears.

**Figure 3:**
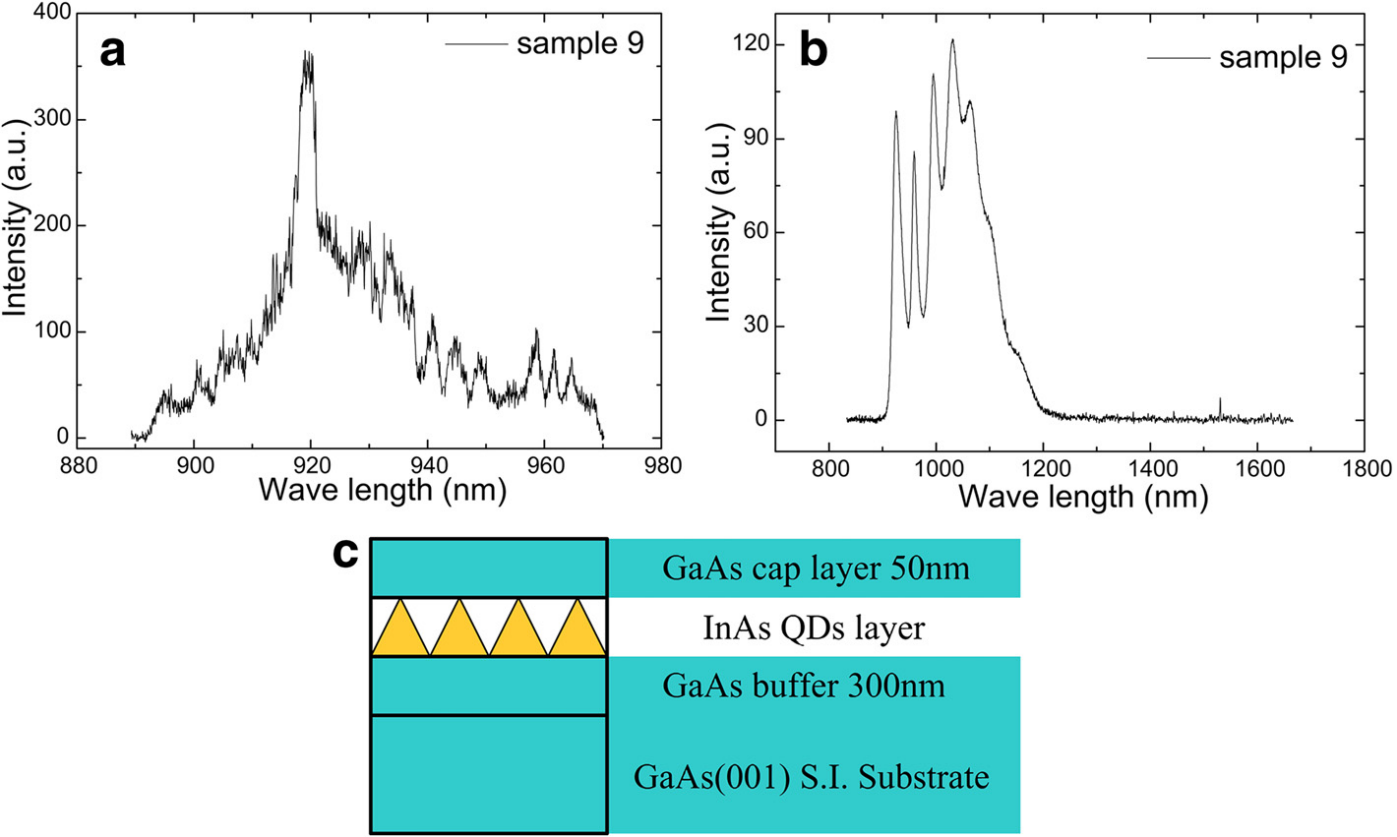
**Micro-PL and Fourier spectrum.** (**a**) Micro-PL of sample 9 at 80 K, (**b**) Fourier spectrum of sample 9 at 80 K, and (**c**) schematic illustration of sample 9.

The density of the InAs QDs is too high for the application of a single-photon source if the deposition of InAs is equal to *θ*_c_ adjusted by the transition of the RHEED pattern from reconstruction streaks to a spotty pattern. According to the kinetic model, the formation of QDs is divided into four steps: atom deposition on the growth surface, adatom diffusion over the surface, attachment and detachment, and 2D-3D growth transition [13]. When the deposited InAs layer was below the critical thickness, as shown in Figure 2a, both main and reconstruction streaky patterns disappeared as described in [14]. Meanwhile, several spots at a fixed position were caused by the transmitted beam. When the spotty pattern appears (Figure 2b), the transformation of the 2D-3D growth has occurred, and the deposition of InAs is defined as the critical thickness (*θ*_c_). For sample 9 (Table 1), the critical thickness (*θ*_c_) of InAs was grown, but the micro-PL and Fourier-PL were envelop curves at 80 K (Figure 3a,b), which demonstrated that the density of QDs was too high for single-photon source devices.

By growing a reference sample to obtain the critical growth parameters, then increasing growth interruption and growth temperature, and decreasing deposition of InAs, a very low density of QDs can be realized [11]. However, the repeatability is very low if the critical conditions were obtained from samples in different batches because of the accidental error and system error, such as differences caused by different molybdenum sample holder blocks, ambience in the growth chamber, measurement of growth rate and temperature, and so on. For our samples used in this method, the repeatability is less than 47%.

**Figure 4:**
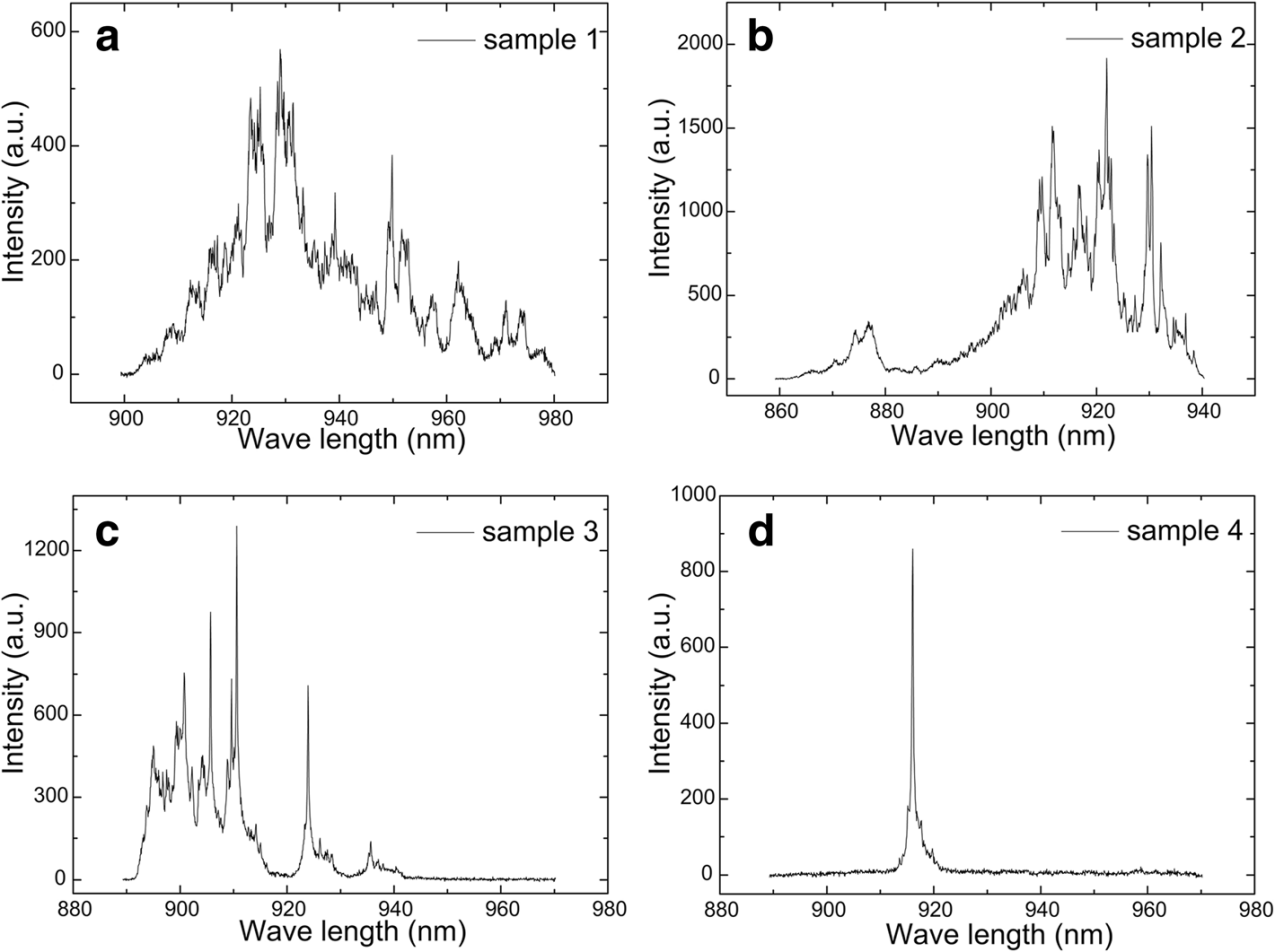
**Micro-PL of samples 1 to 4 at 80 K.** (**a**) Sample 1, △ = 0.15 ML, (**b**) sample 2, △ = 0.075 ML, (**c**) sample 3, △ = 0.025 ML, (**d**) sample 4, △ = 0. △ is the deposition difference between the QD layer and SQD layer.

**Figure 5:**
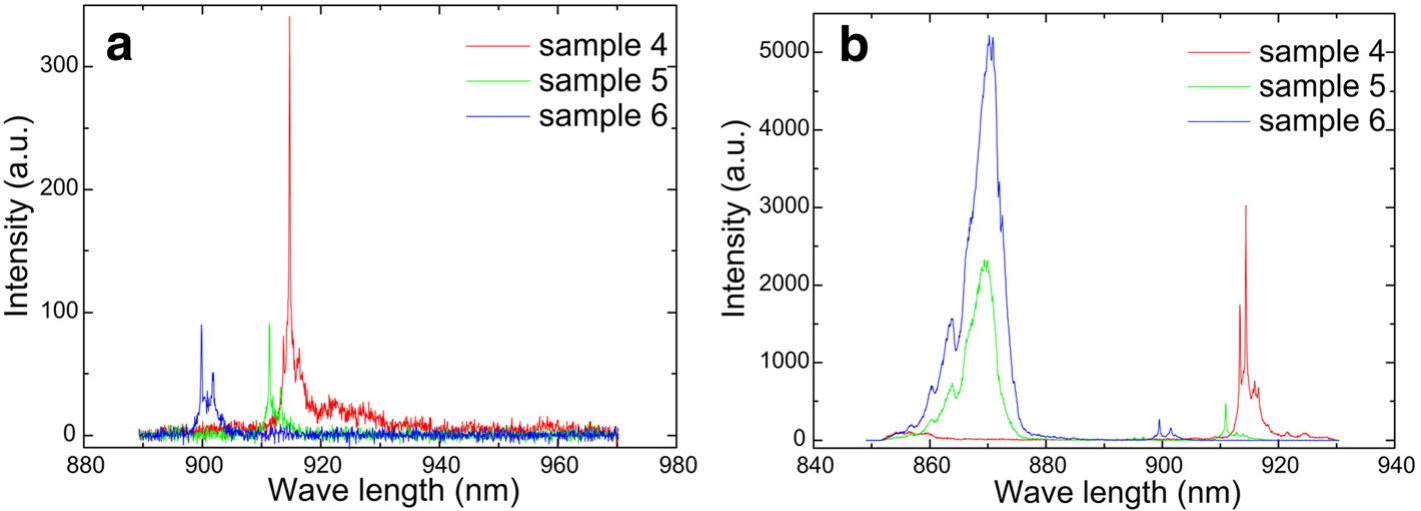
**Micro-PL of samples 4 to 6 at 80 K.** (**a**) Sample 4, △ = 0; sample 5, △ = −0.05 ML; sample 6, △ = −0.075 ML. (**b**) Sample 4, △ = 0; sample 5, △ = −0.05 ML; sample 6, △ = −0.075 ML. △ is the deposition difference between the QD layer and SQD layer.

To resolve this problem, the critical growth parameters were obtained *in situ*. A SQD layer was grown to obtain the *θ*_c_ of InAs QDs and then annealed for the desorption of InAs. After growing a 50-nm GaAs barrier layer to separate the SQD layer, the InAs QD layer was grown to investigate the best condition of low density. Samples 1 to 6 (Table 1) were grown to study the effects of the deposition of InAs. The deposition of the SQD layer was in the critical condition when a spotty pattern just appears. The growth temperature of the QD layer is 5°C higher than that of the SQD layer to achieve lower-density QDs and obtain a better micro-PL spectrum. The spotty pattern in the RHEED did not appear after the growth of the InAs QD layer, which implies that the actual deposition (total deposition − desorption) is slightly less than *θ*_c_. Figures 4 and 5a show a series of micro-PL of decreasing △ from samples 1 to 6. We can find that the micro-PL spectra are multiple lines when △ > 0 and become a sharp single line when △ ≤ 0. As shown in Figure 5a,b, under the same pumping energy, micro-PL transfers from a single narrow peak to double narrow peaks, and the intensity of the spectra decreases sharply. Moreover, blue shift occurs when △ < 0. This can be explained by the fact that QDs are not nucleated completely when deposition is less than the critical condition. In this case, the so-called quantum dots are similar to interface fluctuations. This can also be demonstrated in Figure 5b. When △ < 0, an additional wetting layer peak appears at 870 nm, and the intensity of the peak increases with the decrease of △. We can also find that the micro-PL is sharp and that the peak intensity is highest when △ is equal to 0. Therefore, the best condition of low density is 5°C higher than the growth temperature of the SQD layer, and the deposition of InAs is the same as the SQD layer.

Another reason for the low repeatability is that the condition of the low-density InAs QD for single-photon source devices is strict, so a small deviation of deposition may affect the micro-PL seriously. The micro-PL spectra of samples 3 and 4 at 80 K are shown in Figure 4c,d. The sharp single peak indicates that sample 4 has a good single-photon characteristic. The multiple peaks of sample 3 demonstrate that a slight change (0.025 ML) of deposition may determine the optical characteristic, so the critical growth parameters obtained from the reference sample *ex situ* make the repeatability low.

**Figure 6:**
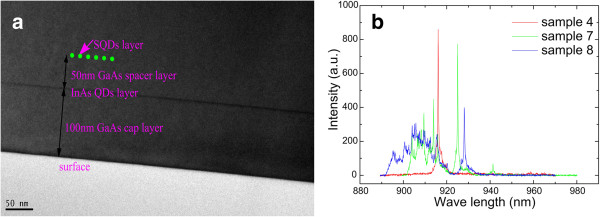
**TEM and micro-PL.** (**a**) TEM of sample 10. (**b**) Micro-PL of samples 4, 7, and 8 annealed respectively at 650°C, 630°C, and 620°C.

The annealing temperature of the SQD layer was also studied. Figure 6a shows the TEM result of sample 10 annealed at 580°C. The green dot line stands at the position of the SQD layer, and the black line is the InAs QD layer. Comparing the InAs QD layer and the SQD layer, it is found that almost all the InAs in the SQD layer desorbed after annealing. However, the micro-PL shows other interesting phenomena in Figure 6b. Firstly, when the annealing temperature decreases, the wavelength increases inversely. This indicates that the InAs SQD layer may be not completely desorbed after annealing. After growth of the 50-nm GaAs barrier layer, the interface roughness of the three samples is different. This results in the larger size of the QD and longer wavelength if the interface is much rougher for samples 7 and 8. Secondly, an additional exciton appears at the shorter wavelength when the annealing temperature of sample 7 decreases. A slight change of the pump laser beam position dramatically restrains the main peak and increases the neighboring multiple peak intensity. This phenomenon is attributed to multiple quantum dots, which demonstrates that the density increases when the annealing temperature decreases. When annealing temperature decreases to 580°C for sample 8, micro-PL becomes a broad emission spectrum. This trend confirms that the interface roughness becomes worse. Therefore, the annealing temperature should not be less than 610°C.

## Conclusion

It is an important issue to accurately control the 2D-3D transition parameters for the growth of low-density self-assembled InAs QDs. We have proposed a method of introducing a sacrificial InAs layer to determine *in situ* the 2D-3D critical condition as a spotty pattern appears in RHEED. After annealing of the InAs sacrificial layer at 610°C, the expected low-density QDs can be grown with highly improved repeatability. As confirmed by micro-PL spectroscopy, high optical-quality low-density QDs were obtained under the growth temperature of 5°C higher than that of the SQD layer and the same deposition of InAs. The slight increase of the InAs deposition amount dramatically deteriorates the PL properties. Our result provides a useful way to accurately control the critical condition of the low-density InAs QDs and thus to improve the fabrication repeatability.

## Abbreviations

θc: Critical coverage; PL: Photoluminescence; QD: Quantum dot; RHEED: Reflection high-energy electron diffraction; SQDs: Sacrificed InAs quantum dots; TEM: Transmission electron microscopy.

## Competing interests

The authors declare that they have no competing interests.

## Authors' contributions

M-FL participated in the design of the study; grew the samples; carried out the TEM images, test of micro-PL, the alignment, and the reconstruction of the data; took part in discussions and in the interpretation of the result; and wrote the manuscript. YY participated in the design of the study, testing of the micro-PL, discussions, and interpretation of the results. J-FH participated in the acquisition of the TEM images and the discussions of the results. YZ and X-jS participated in the discussions of the results. L-JW and H-QN have supervised the writing of the manuscript. H-QN and Z-CN supervised the writing of the manuscript and the experimental part. All the authors have read and approved the final manuscript.

## Authors' information

M-FL, YY, J-FH, L-JW, YZ, and X-jS are Ph.D. students at the Institute of Semiconductors, Chinese Academy of Sciences. H-QN is associate researcher, and Z-CN is a researcher at the State Key Laboratory for Superlattices and Microstructures, Institute of Semiconductors, Chinese Academy of Sciences.
